# pH-Sensitive Nano-Complexes Overcome Drug Resistance and Inhibit Metastasis of Breast Cancer by Silencing Akt Expression: Erratum

**DOI:** 10.7150/thno.42847

**Published:** 2020-01-18

**Authors:** Jieying Yin, Tianqun Lang, Dongmei Cun, Zhong Zheng, Yan Huang, Qi Yin, Haijun Yu, Yaping Li

**Affiliations:** 1 State Key Laboratory of Drug Research & Center of Pharmaceutics, Shanghai Institute of Materia Medica, Chinese Academy of Sciences, Shanghai 201203, China;; 2 School of Pharmacy, Shenyang Pharmaceutical University, Shenyang 110016, China;; 3 University of Chinese Academy of Sciences, Beijing 100049, China.

In the initially published version of this article, the IHC image of the Akt expression of the PMA group in Figure 7E and the lung image of the BMA+PMN group in Figure 9 are wrong. The correct Figure 7E and Figure 9 are as follows:

The corrections made in this erratum do not affect the original conclusions. The authors apologize for any inconvenience or misunderstanding that this error may have caused.

## Figures and Tables

**Figure 7:**
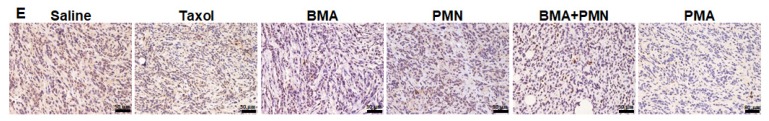
(E) The Akt expression of tumor sections analyzed by IHC. (Scale bar: 50 μm)

**Figure 9:**
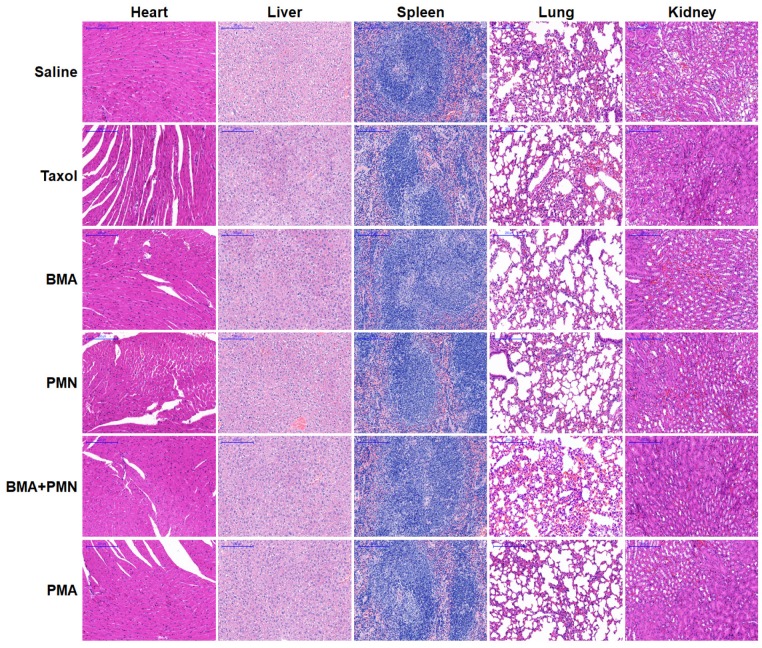
Histopathological analysis in healthy mice following treatment with multiple doses of various formulations. H&E staining images of tissue sections from the mice treated with Saline, Taxol, BMA, PMN, BMA+PMN, and PMA. (Scale bar: 200 μm)
